# Early Hyperglycemia in Pediatric Traumatic Brain Injury Predicts for Mortality, Prolonged Duration of Mechanical Ventilation, and Intensive Care Stay

**DOI:** 10.1155/2015/719476

**Published:** 2015-05-14

**Authors:** Shu-Ling Chong, Sumitro Harjanto, Daniela Testoni, Zhi Min Ng, Chyi Yeu David Low, Khai Pin Lee, Jan Hau Lee

**Affiliations:** ^1^Department of Emergency Medicine, KK Women's and Children's Hospital, 100 Bukit Timah Road, Singapore 229899; ^2^SingHealth Duke-NUS Paediatrics Academic Clinical Programme, 100 Bukit Timah Road, Singapore 229899; ^3^Duke-NUS Graduate Medical School, 8 College Road, Singapore 169857; ^4^Division of Neonatal Medicine, Escola Paulista de Medicina-Universidade Federal de Sao Paulo, Rua Marselhesa 630, Vila Clementino, 04020-060 São Paulo, SP, Brazil; ^5^Department of Paediatric Medicine, KK Women's and Children's Hospital, 100 Bukit Timah Road, Singapore 229899; ^6^Department of Neurosurgery, KK Women's and Children's Hospital, 100 Bukit Timah Road, Singapore 229899; ^7^Children's Intensive Care Unit, KK Women's and Children's Hospital, 100 Bukit Timah Road, Singapore 229899

## Abstract

We aim to study the association between hyperglycemia and in-hospital outcomes among children with moderate and severe traumatic brain injury (TBI). This retrospective cohort study was conducted in a tertiary pediatric hospital between 2003 and 2013. All patients < 16 years old who presented to the Emergency Department within 24 hours of head injury with a Glasgow Coma Scale (GCS) ≤ 13 were included. Our outcomes of interest were death, 14 ventilation-free, 14 pediatric intensive care unit- (PICU-) free, and 28 hospital-free days. Hyperglycemia was defined as glucose > 200 mg/dL (11.1 mmol/L). Among the 44 patients analyzed, the median age was 8.6 years (interquartile range (IQR) 5.0–11.0). Median GCS and pediatric trauma scores were 7 (IQR 4–10) and 4 (IQR 3–6), respectively. Initial hyperglycemia was associated with death (37% in the hyperglycemia group versus 8% in the normoglycemia group, *p* = 0.019), reduced median PICU-free days (6 days versus 11 days, *p* = 0.006), and reduced median ventilation-free days (8 days versus 12 days, *p* = 0.008). This association was however not significant in the stratified analysis of patients with GCS ≤ 8. *Conclusion*. Our findings demonstrate that early hyperglycemia is associated with increased mortality, prolonged duration of mechanical ventilation, and PICU stay in children with TBI.

## 1. Background

Traumatic brain injury (TBI) remains a significant health burden worldwide [1, 2]. First-line physicians are particularly interested in predictors for severe injury. This is especially relevant in pediatrics, due to the varied and nonspecific complaints among head-injured children. Age, Glasgow Coma Scale (GCS), accidental hypothermia, hyperglycemia, and coagulation disorders are reported to be independent prognostic factors for mortality [3].

Among adults with TBI, studies demonstrated an association between hyperglycemia and poor neurological outcomes [4–7]. Similarly, hyperglycemia prognosticated children with TBI [8–11]. Specifically, a recent paper demonstrated that severe hyperglycemia (>200 mg/dL {11.1 mmol/L}) was associated with poor Glasgow Outcome Scores when compared with mild blood glucose elevation (110–160 mg/dL {6–9 mmol/L}) [12]. Furthermore,* persistent* hyperglycemia beyond the initial presentation was associated with poorer neurological outcomes [13] and death [14]. However, this association is not consistent; other investigators have demonstrated that hyperglycemia associated with pediatric closed head injury was transient and did not predict patient outcomes [15].

Previous studies have focused primarily on mortality as their outcome of interest. Furthermore, there are only limited published studies in this area in Asia [16]. To add to the evidence provided by previous investigators, we were specifically interested in examining the impact of hyperglycemia on in-hospital outcome measures such as duration of mechanical ventilation, length of pediatric intensive care unit (PICU), and hospital stay in children with TBI. We performed a study to examine the association between hyperglycemia and clinical outcomes in children with moderate (GCS 9–13) and severe TBI (GCS ≤ 8). We hypothesized that hyperglycemia, in children with moderate to severe TBI, is associated with death, prolonged duration of mechanical ventilation, PICU, and hospital stay.

## 2. Methods

### 2.1. Study Design and Setting

We conducted a retrospective cohort study in KK Women's and Children's Hospital, Singapore. Our hospital is a large tertiary center that sees about 28,000 trauma patients at the emergency department (ED) annually. The majority of these comprise minor trauma. We traced all records coded under the* International Classification of Diseases (ICD)* diagnosis of head injury with a GCS of ≤13. All patients < 16 years of age who presented to the ED over the period from 2003 to 2013, with GCS ≤ 13, were included. Patients were excluded if they were ≥ 16 years, received prior treatment at another hospital and were transferred > 24 hours after the head injury, were drowsy from other causes (apart from the head injury), or did not have any glucose levels documented in the ED or PICU. Children with moderate to severe TBI in our institution are routinely maintained with head up nursing (once the cervical spine is cleared), normocarbia, strict temperature control and we utilize hyperosmolar therapy when indicated. Morphine and midazolam are routinely used for analgesia and sedation, whereas paralysis is reserved for cases with high intracranial pressure (ICP). During the 10-year period, mannitol was replaced with 3% hypertonic saline as the choice hyperosmolar agent. Secondly, where cooling was previously attempted in the past, we now aim for normothermia for the past 2-3 years following current evidence [17, 18]. Throughout the 10-year period, second tier management included the use of thiopentone and, in some cases, decompressive craniectomy.

Our local institutional review board approved this study without the need for informed consent.

### 2.2. Variables

Demographic details of each patient were collected. We reviewed presenting glucose levels at the ED, and subsequent peak values recorded in the PICU at 0–24 hours, 24–48 hours, and 48–72 hours. We defined hyperglycemia as glucose > 200 mg/dL (11.1 mmol/L) [13]. As part of a sensitivity analysis, we repeated our analysis using a different glucose threshold for hyperglycemia: 150 mg/dL (8.3 mmol/L) [13]. We also collected data on the mechanism of injury, presenting GCS, presence of polytrauma, as well as pediatric trauma scores (PTS). We divided presenting GCS into two groups: GCS ≤ 8 and GCS > 8. The PTS is a measure of injury severity, as measured by the weight of the child, the airway status, systolic blood pressure, level of consciousness, and the type and complexity of fractures or wounds present [19, 20].

### 2.3. Outcome Measures

Our primary outcome was hospital mortality. Secondary outcomes were duration of mechanical ventilation, PICU, and hospital length of stay (LOS). To account for death as a competing outcome, we summarized the requirement for mechanical ventilation as ventilator-free days with a maximum of 14 days. For LOS, we utilized PICU-free and hospital-free days with a maximum of 14 and 28 days, respectively.

### 2.4. Statistical Methods

Data were summarized and reported in the following way: Continuous variables were presented as medians (with interquartile ranges (IQR)), while binary or categorical data was presented as numbers and frequencies (%). Univariate statistical tests for binary or categorical variables were conducted by chi-square test, while continuous variables were assessed by the Mann-Whitney test. We performed a separate stratified analysis of children with GCS ≤ 8. Statistical significance was taken as *p* values < 0.05 for all tests. Statistical analysis was performed using Stata 12.1 (College Station, TX).

## 3. Results

**Figure 1:**
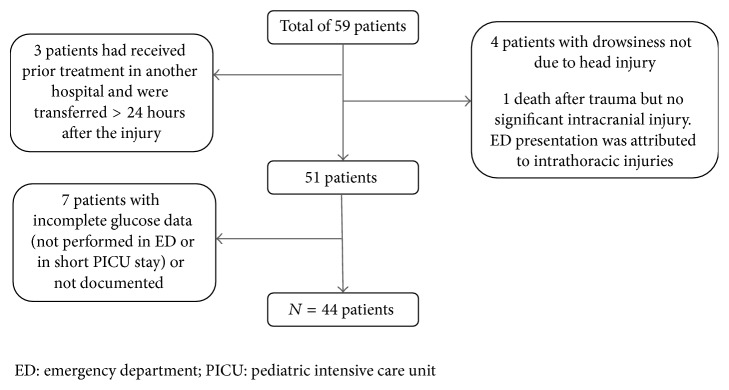
Flow chart of cohort of children with moderate to severe traumatic brain injury.

Out of a total of 59 patients who matched the inclusion criteria, three were excluded because of prior care in other institutions and had a delayed transfer of >24 hours to our hospital. Five were found to have drowsiness attributed to causes other than the head injury and seven patients had incomplete data; they did not have glucose measurements in the ED or during the PICU stay (Figure 1). Of the 44 patients with complete data, the median age was 8.6 years (IQR 5.0–11.0), with a median GCS of 7 (IQR 4–10) and a median PTS of 4 (IQR 3–6) (Table 1). Three patients who were transferred from other local institutions had a median delay of 5 hours between the time of injury and time of arrival in our center. The most common mechanism of injury was by motor vehicle accidents (22 patients, 50.0%). 21 (47.7%) patients had polytrauma. The overall mortality was 9/44 (20.5%). There were 16 patients (36.4%) who sustained skull and base of skull fractures, 36 (81.8%) with focal intracranial bleeds (not exclusive: SDH (16, 44.4%), EDH (13, 36.1%), SAH (8, 22.2%), and intraparenchymal bleed (8, 22.2%). 20 (45.5%) patients had cerebral edema and 7 (15.9%) had diffuse axonal injury.

Thirty-one (70.5%) patients underwent neurosurgical interventions. Of these, the majority (30 patients, 96.8%) underwent insertion of extraventricular catheter for ICP monitoring, while 15 patients (48.4%) underwent evacuation of intracranial clots. Among the 44 patients, we only had documented ICP and cerebral perfusion pressure (CPP) values for 23 patients. On the first day after admission, the median of the highest ICP was 33 mmHg (IQR 24–45). The highest and lowest CPP values were 93 mmHg (IQR 86–105) and 40 mmHg (IQR 20–48), respectively.

**Figure 2:**
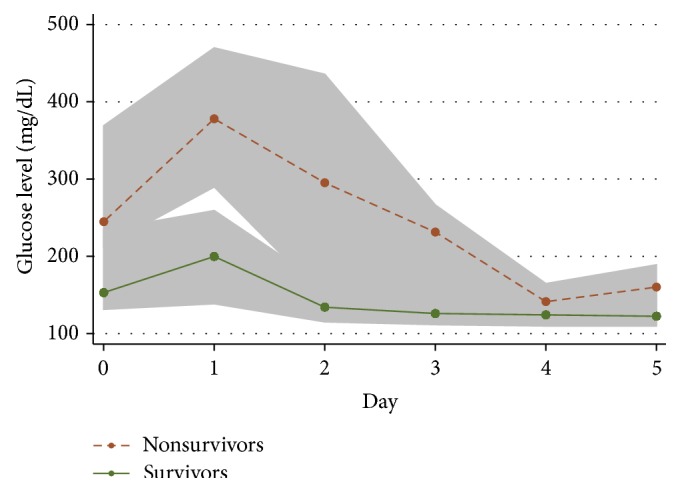
Glucose levels for survivors versus nonsurvivors over time. The points signify the median glucose levels and the shaded area signifies the interquartile range.

The median glucose in ED was 180.0 mg/dL {10 mmol/L} (IQR 131.4–257.4 mg/dL {7.3–14.3 mmol/L}). Two patients died in the ED. Both patients arrived with a GCS of 3 and had hypoglycemia on arrival (45 mg/dL {2.5 mmol/L} and 18 mg/dL {1 mmol/L}). Among the 42 patients who survived to admission, only five patients (11.9%) received insulin infusion at 0–24 hours of admission, and four of these (9.5%) continued to receive insulin infusion at 24–48 hours of hospitalization. Fourteen of our patients (31.8%) received inotropes. These consisted of dopamine, adrenaline, and noradrenaline, in varying combinations. The other 7 deaths occurred between the 3rd and 10th day of stay in the PICU. The separation of glucose levels between survivors and nonsurvivors was most marked at about 24 hours after arrival in the ED (Figure 2). Only 27 patients (61.4%) had documented lactate levels (in the first 24 hours after arrival) because blood lactate was instituted only after the starting date of this retrospective study. The median peak level of lactate in the first 24 hours among these 27 patients was 2.6 mmol/L (IQR 1.7–4.8 mmol/L).

Univariate analysis showed that age, GCS on presentation, presence of hyperglycemia, PTS (pediatric trauma score), and mechanism of injury were significantly associated with death (Table 1). There was a significant difference for death, 14 PICU-free and 14 ventilation-free days between those with hyperglycemia and those with normoglycemia at the ED (Table 2). Excluding the two patients that died in the ED (both had hypoglycemia), seven out of the 19 (36.8%) patients with initial hyperglycemia died, but no death was observed among the remaining 23 patients with normoglycemia (*p* = 0.019). Using a cutoff glucose of 150 mg/dL (8.3 mmol/L), hyperglycemia remained statistically significant for prolonged duration of mechanical ventilation and PICU stay (Table 2). Persistent hyperglycemia up to 48 and 72 hours was also significantly associated with increased risk of death (83.3% in hyperglycemia group versus 5.6% in normoglycemia group at 48 hours, and 80% versus 5.6% at 72 hours, resp., *p* < 0.001).

We went on to perform a stratified analysis of patients with GCS ≤ 8. The effect of hyperglycemia on the above clinical outcomes was not significant (Table 3) at both cutoff values of 200 mg/dL (11.1 mmol/L) and 150 mg/dL (8.3 mmol/L).

There were three cases of nonaccidental injury (NAI) in our group. The first two patients who were finally diagnosed with subdural hemorrhage and died in hospital were both 3 years old. The third patient was a 1-year-old boy who presented with sudden onset of drowsiness and decreased movement, diagnosed with a right frontoparietal subdural hemorrhage. This child was discharged with recurrent focal seizures and required physiotherapy on follow-up. The first two patients had hyperglycemia on presentation as well as persistent hyperglycemia up to 48 hours.

On following up the patients in their course of PICU stay, 9 patients (20.4%) had febrile illness, associated with bacteriological confirmation or clinical signs of sepsis; most of these were secondary to pneumonias or urinary tract infections.

## 4. Discussion

Our study among pediatric TBI patients showed that admission hyperglycemia has a significant association with poor clinical outcomes of death, duration of mechanical ventilation, and PICU LOS. However, stratified analysis of patients with GCS ≤ 8 did not demonstrate an association between hyperglycemia and these clinical outcomes.

Hyperglycemia (both peak glucose and persistent hyperglycemia) has been shown to be an independent predictor for mortality among ill children in the PICU [21]. There are multifactorial causes of hyperglycemia consistent with the pathophysiological stress response after injury. The release of catecholamine and cortisol, together with glucose intolerance, has been documented in patients with head injury [22–24]. Other studies have demonstrated that hyperglycemia worsens the impact of ischemia and hypoxia, leading to worse outcomes [25, 26]. In addition, glucose metabolism changes significantly after TBI, as evident by the presence of mitochondrial dysfunction and increased cerebral utilization of glucose [27–29].

Serum glucose has previously been reported to be a prognostic factor in severe TBI (Table 4). In particular, high admission glucose (≥200 mg/dL {11.1 mmol/L}) has been demonstrated to be associated with poor outcomes, in both adults and children [4–12]. A proposed scale to be applied during the first hours after hospital admission in children with severe TBI included glucose as an independent predictor for death [3]. Our study findings were consistent with the current literature showing that the presence of hyperglycemia is associated with poor outcomes. In addition to increased mortality and poorer neurological outcomes, our study demonstrated that head-injured children with hyperglycemia also tend to have longer periods of mechanical ventilation and PICU stay. However, when we accounted for severity of injury using a stratified analysis, this effect was not significant. This lack of statistical association could be attributed to the small numbers in our study. We were not able to perform a multivariate analysis on the above predictors due to the small population size.

Randomized controlled trials in critically ill pediatric populations (not limited to TBI) have focused on the effect of tight glycemic control. One randomized controlled trial of critically ill children showed an improvement in inflammatory markers and reduced length of ICU stay in patients with tight glycemic control but also an increased risk of hypoglycemia [30]. However, a recent randomized trial among children admitted to the PICU showed no significant difference in the number of days alive and free from mechanical ventilation at 30 days after randomization, with the incidence of hypoglycemia being higher in the tight glucose control group compared to that with conventional glucose control [31]. Both of these studies did not describe in detail the subgroup of children with TBI and their corresponding severity. A study looking specifically at TBI patients would be able to better answer the question of whether glucose control may modify outcomes in pediatric TBI. Besides the peak and duration of hyperglycemia, glucose variability has also been shown to increase the risk of mortality, and any intervention should address this variability as well [32].

Focusing on other possible prognostic factors following pediatric TBI, we also found that the median PTS was significantly associated with mortality (Table 1). A previous report of 180 patients [33] demonstrated that patients with serious concomitant injuries had fewer days off mechanical ventilation and longer hospital stay, although it did not significantly predict for mortality. In the above study, patients with serious concomitant injuries were most likely involved in motor vehicle accidents and had more severe TBI [33]. In another study of 100 adults with TBI, high grade concomitant injuries were associated with functional disability 18 months after the injury [34].

We looked specifically at our patients with NAI because this is a special subset in the vulnerable pediatric population. It has been previously reported that GCS and PTS both predict for mortality among children with NAI [35]. The glycemic response in these children may also differ from other types of TBI, evident from a previous report that inflicted TBI patients displayed a 14% reduction in peak stimulated cortisol compared with accidental TBI patients [36]. In another study focusing on inflicted traumatic brain injury among 35 children [37], initial hyperglycemia was found to be significantly associated with a poorer Glasgow Outcome Scale score. However, when controlled for age at injury and time since injury on multivariate analysis, the association was not significant. Two out of our three patients with NAI had hyperglycemia on presentation that persisted for up to 48 hours.

We recognize the following limitations in our study: (1) Small numbers: this was due to a low prevalence of moderate and severe TBI in our population. We look forward to accrual of more data with a prospective surveillance registry. (2) Over the period in which we utilized the data, there may be unmeasured differences in clinical management with regard to ventilator management and intracranial pressure control strategies, both of which may potentially affect the clinical outcomes of interest in our study. These changes include moving from the use of mannitol to 3% hypertonic saline as the preferred hyperosmolar agent and maintaining normothermia instead of instituting hypothermia for patients with TBI. Other aspects of first and second tier management have not otherwise changed significantly. (3) There was no uniform protocol guiding the use of insulin for persistent hyperglycemia throughout this study period.

## 5. Conclusion

Hyperglycemia, in children with moderate and severe TBI, is associated with mortality, prolonged duration of mechanical ventilation, and longer duration of PICU stay. Our findings build on the current evidence that in-hospital outcomes are adversely associated with hyperglycemia. Our findings are part of an ongoing study to derive prognostic factors for pediatric TBI in our center. To address the question of whether glucose control impacts clinical outcomes in children with TBI, we propose that future research on glucose homeostasis in critically ill children focuses specifically on children with moderate and severe TBI.

## Figures and Tables

**Table 1:** Clinical demographics and mechanism of injury for both survivors and nonsurvivors with moderate and severe traumatic brain injury.

	All (*n* = 44)	Survivors (*n* = 35)	Nonsurvivors (*n* = 9)	*p* value
Age (years), median (IQR^a^)	8.6 (5.0–11.0)	9.2 (6.4–11.4)	3.9 (2.1–5.8)	0.037
Gender, males, *n* (%)	28 (64)	22 (63)	6 (67)	1.000
GCS^b^ ≤8 on admission, *n* (%)	23 (52)	14 (40)	9 (100)	0.002
Presence of polytrauma, *n* (%)	21 (48)	15 (43)	6 (67)	0.202
Initial glucose, mg/dL, median (IQR^a^)	180 (131–257)	153 (131–234)	245 (212–369)	0.116
Initial glucose >200 mg/dL, *n* (%)	19 (43)	12 (34)	7 (78)	0.027
Pediatric trauma score, median (IQR^a^)	4 (3–6)	4 (4–7)	1 (0–3)	0.001
Mechanism of injury, *n* (%)				0.048
Road traffic accident	22 (50)	18 (51)	4 (44)
Fall	15 (34)	14 (40)	1 (11)
Nonaccidental injury	3 (7)	1 (3)	2 (22)
Others	4 (9)	2 (6)	2 (22)
Number requiring neurosurgery, *n* (%)	31 (71)	26 (74)	5 (56)	0.272

^a^IQR: interquartile range; ^b^GCS: Glasgow Coma Score.

**Table 2:** Presenting glucose as a predictor for clinical outcomes in patients with GCS ≤13.

Hyperglycemia defined as glucose >200 mg/dL (11.1 mmol/L)	ED^a^ normoglycemia (*n* = 25)	ED^a^ hyperglycemia (*n* = 19)	*p* values
Death, *n* (%)	2 (8)	7 (37)	0.019
28 hospital-free days, median (IQR^b^)	21 (6–23)	11 (0–20)	0.222
14 pediatric intensive care unit-free days, median (IQR^b^)	11 (7–12)	6 (3–10)	0.006
14 ventilation-free days, median (IQR^b^)	12 (8–13)	8 (4–10)	0.008

Hyperglycemia defined as glucose >150 mg/dL (8.3 mmol/L)	ED^a^ normoglycemia (*n* = 19)	ED^a^ hyperglycemia (*n* = 25)	*p* values

Death, *n* (%)	2 (11)	7 (28)	0.155
28 hospital-free days, median (IQR^b^)	21 (7–23)	18 (0–23)	0.273
14 pediatric intensive care unit-free days, median (IQR^b^)	11 (7–12)	6 (3–10)	0.032
14 ventilation-free days, median (IQR^b^)	12 (8–13)	8 (4–11)	0.021

^a^ED: emergency department; ^b^IQR: interquartile range.

**Table 3:** Presenting glucose as a predictor for clinical outcomes in patients with GCS ≤8 (*n* = 28).

Hyperglycemia defined as glucose >200 mg/dL (11.1 mmol/L)	ED^a^ normoglycemia (*n* = 13)	ED^a^ hyperglycemia (*n* = 15)	*p* values
Death, *n* (%)	2 (15)	7 (47)	0.077
28 hospital-free days, median (IQR^b^)	5 (0–23)	10 (0–23)	0.587
14 pediatric intensive care unit-free days, median (IQR^b^)	7 (0–12)	5 (2–10)	0.815
14 ventilation-free days, median (IQR^b^)	8 (0–13)	7 (2–10)	0.889

Hyperglycemia defined as glucose >150 mg/dL (8.3 mmol/L)	ED^a^ normoglycemia (*n* = 9)	ED^a^ hyperglycemia (*n* = 19)	*p* values

Death, *n* (%)	2 (22)	7 (37)	0.439
28 hospital-free days, median (IQR^b^)	5 (0–7)	10 (0–23)	0.668
14 pediatric intensive care unit-free days, median (IQR^b^)	7 (0–8)	5 (0–10)	0.960
14 ventilation-free days, median (IQR^b^)	8 (0–9)	7 (2–11)	0.901

^a^ED: emergency department; ^b^IQR: interquartile range.

**Table 4:** Previous studies reporting on hyperglycemia as a predictor for poor outcomes in pediatric traumatic brain injury.

Study	Design	Inclusion criteria (total *n*)	Results	Comments
Melo et al. [3, 10]	Retrospective cross-sectional	Children with severe TBI (GCS ≤8). Mean age of 7 years (*n* = 315).	Hyperglycemia ≥200 mg/dL is an independent predictor for mortality—OR 6.14 (95% CI 2.25–16.73).	A new scale was proposed; this included age group, GCS, temperature, blood glucose levels, and prothrombin time.

Cochran et al. [9]	Retrospective review	Children admitted with a head regional AIS ≥3. Median age of 4 years (*n* = 170).	Admission glucose had adjusted OR for head-injury related death of 1.01 (95% CI 1.003–10.23).	On multivariate analysis, GCS was also an independent predictor for head-injury related death.

Smith et al. [13]	Retrospective review of a prospectively collected pediatric neurotrauma registry	Children admitted with severe TBI (GCS ≤8). Mean age of 81 months (*n* = 57).	Mean glucose concentrations in the early period (<48 hours) were similar in children with favorable and unfavorable outcomes. Hyperglycemia in the late period (49–168 hours) was associated with unfavorable GOS at 6 months.	As part of the protocol, if serum glucose ≥70 mg/dL, glucose administration was avoided for 48 hours after TBI.

Seyed Saadat et al. [14]	Retrospective cross-sectional	Children with severe TBI (GCS ≤8), admitted to ED within 12 hours of injury. Median age of 13 years (*n* = 122).	Persistent hyperglycemia during the first 2 and first 3 days had adjusted ORs for mortality of 2.84 (95% CI 0.89–9.06) and 11.11 (95% CI 2.95–41.71), respectively.	Persistent hyperglycemia is an independent predictor of mortality.

Elkon et al. [12]	Retrospective cohort	Children with moderate (GCS 9–12) and severe TBI (GCS 3–8). Mean age (of severe hyperglycemia group) of 6.1 years (*n* = 271).	Severe blood glucose elevation (blood glucose >200 mg/dL) had increased adjusted OR of 3.5 for poor GOS, compared with mild glucose elevation (glucose 110–160 mg/dL).	Severe blood glucose elevation was independently associated with poor outcome.

Parish and Webb [15]	Retrospective case control	Children admitted with GCS of 3–10, between 24 months and 12 years. Mean age of (of cases) 7 years (*n* = 36, 37 controls).	The hyperglycemic response was more common among those with head trauma (40% compared to controls (5%) but within the head trauma group, the level of hyperglycemia was not associated with death or need for extended care).	GCS on admission was a significant prognostic indicator. In this study, authors conclude that the hyperglycemia is transient and does not warrant treatment with insulin.
